# Associations between bacterial and fungal communities in the human gut microbiota and their implications for nutritional status and body weigh﻿t

**DOI:** 10.1038/s41598-024-54782-7

**Published:** 2024-03-08

**Authors:** Ricardo García-Gamboa, Osiris Díaz-Torres, Carolina Senés-Guerrero, Misael Sebastián Gradilla-Hernández, Andrés Moya, Vicente Pérez-Brocal, Alejandro Garcia-Gonzalez, Marisela González-Avila

**Affiliations:** 1 Centro de Investigación y Asistencia en Tecnología y Diseño del Estado de Jalisco A.C., Av. Normalistas No. 800, col Colinas de la Normal, 44270 Guadalajara, Jalisco Mexico; 2 Tecnologico de Monterrey, Escuela de Medicina y Ciencias de la Salud, Av. General Ramon Corona 2514, Nuevo Mexico, 45138 Zapopan, Jalisco Mexico; 3 Tecnologico de Monterrey, Escuela de Ingenieria y Ciencias, Laboratorio de Sostenibilidad y Cambio Climático, Av. General Ramon Corona 2514, 45138 Zapopan, Jalisco Mexico; 4 Department of Genomics and Health, Foundation for the Promotion of Health and Biomedical Research of Valencia Region (FISABIO-Public Health), Valencia, Spain; 5 CIBER in Epidemiology and Public Health (CIBEResp), Madrid, Spain; 6 Institute for Integrative Systems Biology (I2SysBio), The University of Valencia and The Spanish National Research Council (CSIC-UVEG), Valencia, Spain

**Keywords:** Gut mycobiota, Gut bacteriota, Obesity, Gut dysbiosis, Bacillota/Bacteroidota (Firmicutes/Bacteroidetes) ratio, *Candida*, Biotechnology, Microbiology, Molecular biology, Health care

## Abstract

This study examined the interplay between bacterial and fungal communities in the human gut microbiota, impacting on nutritional status and body weight. Cohorts of 10 participants of healthy weight, 10 overweight, and 10 obese individuals, underwent comprehensive analysis, including dietary, anthropometric, and biochemical evaluations. Microbial composition was studied via gene sequencing of 16S and ITS rDNA regions, revealing bacterial (bacteriota) and fungal (mycobiota) profiles. Bacterial diversity exceeded fungal diversity. Statistically significant differences in bacterial communities were found within healthy-weight, overweight, and obese groups. The Bacillota/Bacteroidota ratio (previously known as the Firmicutes/Bacteroidetes ratio) correlated positively with body mass index. The predominant fungal phyla were Ascomycota and Basidiomycota, with the genera *Nakaseomyces*, *Kazachstania*, *Kluyveromyces*, and *Hanseniaspora*, inversely correlating with weight gain; while *Saccharomyces*, *Debaryomyces*, and *Pichia* correlated positively with body mass index. Overweight and obese individuals who harbored a higher abundance of *Akkermansia muciniphila,* demonstrated a favorable lipid and glucose profiles in contrast to those with lower abundance. The overweight group had elevated *Candida*, positively linked to simple carbohydrate consumption. The study underscores the role of microbial taxa in body mass index and metabolic health. An imbalanced gut bacteriota/mycobiota may contribute to obesity/metabolic disorders, highlighting the significance of investigating both communities.

## Introduction

The gastrointestinal tract harbors a complex and diverse community of microorganisms collectively known as the gut microbiota, which plays pivotal roles in human physiology and pathophysiology^1^. While bacteria dominate this niche, it also hosts other microorganisms, including archaea, fungi and yeasts. Viruses also inhabit this environment^2^. Distinct microbial assemblages populate the intestines, with the bacterial community referred to as the intestinal bacteriota (IBac) and the fungal community as the intestinal mycobiota (IMy)^3^. The exploration of gut microbial composition is of paramount importance for comprehending human health and disease states. The IBac has been linked to the etiology of obesity and establishes symbiotic relationships with the human body, participating in key physiological processes such as digestion and metabolism, thereby enhancing energy extraction from dietary components^4^. Additionally, the IBac contributes to the development of low-grade inflammation, which may underlie obesity and metabolic disorders^5^. With approximately 39% of adults worldwide classified as overweight and 13% as obese, obesity poses significant global health challenges^6^, and it is associated with a range of pathologies, including cardiovascular diseases, metabolic syndrome, and cancer^7^. Consequently, gaining an insight into intestinal microbiota and modulating it are crucial for unraveling the progression of obesity^8^. Furthermore, certain microorganisms in the gut have been linked to susceptibility to inflammatory bowel diseases such as Crohn's disease and ulcerative colitis^9^. The dominant bacterial phyla in the gut are Bacillota and Bacteroidota^10^, formerly referred to as Firmicutes and Bacteroidetes, respectively^11^. The ratio of Bacillota to Bacteroidota shows a positive correlation to body mass index (BMI)^12–14^. In contrast to the well-studied IBac, the IMy remains relatively unexplored. Ascomycota and Basidiomycota are the main phyla detected in culture-based IMy studies, with Ascomycota prevailing as the most abundant^15^. Similar findings were observed in a sequencing study of IMy in obese subjects, employing the Internal Transcribed Spacer (ITS) rDNA region^16^. Among the fungal genera identified in the IMy are *Saccharomyces*, *Malassezia*, and *Candida*^17^. The advent of high-throughput sequencing has enabled comprehensive analyses of both the taxonomic composition and functional attributes of the gut microbiota through omics techniques^18^. There is a compelling need for comprehensive research to shed light on the microbial composition of both IBac and IMy and their intricate associations with various human physiological and pathological states. The objective of this study is to assess the composition of the intestinal bacteriota and mycobiota and explore their correlation with nutritional aspects in relation to obesity.

## Materials and methods

### Subjects

The present study examined a group of 30 Mexican adult participants, who were categorized into three distinct cohorts based on their body mass index (BMI). Specifically, Group 1 was composed of 10 healthy-weight individuals, exhibiting BMIs ranging from 19.5 to 24.9; Group 2 comprised 10 overweight individuals, displaying BMIs between 25.0 and 29.9. Lastly, Group 3 comprised 10 obese individuals, with BMIs in the range of 30.0–34.9. The eligibility criteria for inclusion in the study required that participants were aged between 20 and 50 years, free from any history of intestinal, diabetic, or metabolic disorders, and refrained from the consumption of antibiotics, antifungals, probiotics, or prebiotics for a minimum period of three months leading up to the study. All participants signed informed consent forms prior to their participation.

### Dietary analysis

To assess the dietary patterns of the participants, quantitative questionnaires were employed. Initially, a food-frequency questionnaire was administered to elicit information regarding their habitual food consumption over an extended period (6 months). Participants were required to report both the frequency and specific quantities of the foods they ingested. Subsequently, a 24 h dietary recall was conducted to capture precise quantitative data on the nutritional intake during the preceding 24 h. Furthermore, the data retrieved from the dietary questionnaires completed by each participant provided comprehensive insights into their daily caloric and macronutrient (e.g., carbohydrates, proteins, and fats) intake. The data was subsequently utilized to evaluate and juxtapose the nutritional adequacy of the participants' diets and their potential correlation with the study's outcomes^15^.

### Body composition assessment

The present study carried out an anthropometric evaluation to gain comprehensive insights into the body composition of the participants. The assessment encompassed the use of precise equipment, including a digital scale (model UM-040; TANITA) with a maximum capacity of 150 kg and a grading precision of 100 g; an adipometer (Slim Guide) graded in millimeters; and a metallic flexible measuring tape, 0.5 cm in width, and graded in centimeters and centimeter decimals (Cescorf). The anthropometric measurements conducted on each participant included weight (kg); height (cm); skinfold measurements at the tricipital, bicipital, iliac crest, and subscapular regions (mm); as well as circumferences of the hip, waist, arm, and wrist (cm). All measurements were executed in strict accordance with the criteria prescribed by the International Society for the Advancement of Kinanthropometry (ISAK)^19^.

### Analysis of biochemical parameters

In order to assess comprehensively the biochemical profile of the participants, a thorough blood analysis was conducted. The examination encompassed an evaluation of the following constituents: total iron-binding capacity (TIBC), serum Fe, ferritin, transferrin, albumin, globulin, total bilirubin, direct bilirubin, indirect bilirubin, total proteins, cholesterol, high-density lipoprotein (HDL), low-density lipoprotein (LDL), very low-density lipoprotein (VLDL), triglycerides, glucose, ureic nitrogen, creatinine, uric acid, alanine aminotransferase (ALT), aspartate aminotransferase (AST), glutamic gamma transferase (GGT), and lactate dehydrogenase (LDH). These precise biochemical parameters were instrumental in appraising the nutritional status and metabolic well-being of the participants^15^.

### Microbial analysis

#### DNA extraction

For the comprehensive microbial analysis, fecal samples were collected from each of the 30 participants. All of them received specific instructions on the precise sampling procedure, and the appropriate storage of samples at a temperature of 4 °C, ensuring their integrity upon delivery to the laboratory. The collected stool samples were expeditiously frozen at − 80 °C, thus ensuring optimal preservation until subsequent analyses. Fecal samples of 180–220 mg were used for the extraction of bacterial and fungal DNA using the QIAamp® DNA Stool Mini Kit extraction kit, following the manufacturer's prescribed protocols.

#### Library construction and sequencing

The taxonomic identification of bacteria relied on the sequences derived from the V3 and V4 variable regions of the 16S rDNA gene. To construct the libraries, the Illumina protocol (16S Metagenomic Sequencing Library Preparation) (Cod. 15044223 Rev. B) was followed. Initially, microbial genomic DNA was stored at a concentration of 5 ng/μL in 10 mM Tris pH 8.5. Subsequently, fluorometric quantification using the Qubit fluorometer (ThermoFisher Scientific) ensured accurate adjustments to the DNA concentration. The DNA was then subjected to amplification using specific primers targeting the V3–V4 regions of the 16S rRNA gene^20^, followed by ligation to an adapter, which facilitated the multiplexing step through the incorporation of indices to distinguish individual samples via the Nextera XT Index Kit (FC-131-1096). Prior to sequencing, the library was quantified once again using the Qubit fluorometer. Sequencing was carried out with 2 × 300 bp paired-end reads (MiSeq v3 reagent kit (MS-102-3001)) on a MiSeq sequencer, following the manufacturer's guidelines (Illumina). For fungi and yeasts, the taxonomic identification relied on reads obtained from the ITS1 rRNA gene. The library preparation protocol remained consistent, with specific modifications involving the utilization of distinct primers. For bacteria, the primers utilized were: Forward primer 5′-TCGTCGGCAGCGTCAGATGTGTATAAGAGACAGCCTACGGGNGGCWGCAG-3′ and reverse primer 5′- GTCTCGTGGGCTCGGAGATGTGTATAAGAGACAGGACTACHVGGGTATCTAATCC-3′, while for fungi, the primers used were: Forward primer 5′-TCGTCGGCAGCGTCAGATGTGTATAAGAGACAG-GATGAAGAACGYAGYRAA -3′ and reverse primer 5′-GTCTCGTGGGCTCGGAGATGTGTATAAGAGACAG-TCCTCCGCTTWTTGWTWTGC-3′^21^. Following library quantification, sequencing ensued with a 2 × 300 bp paired-end reads (MiSeq v3 reagent kit (MS-102-3001)) on a MiSeq sequencer, adhering to the manufacturer's instructions (Illumina).

### Bioinformatic analysis

Gut microbiota composition, relative abundance, and ecological diversity were analyzed using QIIME 2.0 software (Quantitative Insights Into Microbial Ecology)^22^. The DADA2 algorithm efficiently filtered chimeric sequences, marginal errors, and noisy reads, resulting in reliable amplicon sequence variants (ASVs)^23^. Denoise-paired processing was applied separately to bacterial and fungal sequences. Bacterial sequences were truncated at 250 bp (forward) and 220 bp (reverse) with trim lengths of 17 bp (forward) and 21 bp (reverse) using 8 threads. For fungal sequences, truncation lengths of 280 bp (both forward and reverse) and trim lengths of 18 bp (forward) and 20 bp (reverse) were applied with 8 threads. Feature tables (FeatureData(Sequence) and FeatureData(Taxonomy)) were generated at 99% identity against the SILVA 132 database^24^ for the 16S rRNA gene and the UNITE database for the ITS1 region^25^. Prior to taxonomic assignment, human sequences were filtered out using bowtie2 (version 2.3.4.2) against the GRCh38.p13 human reference database. Subsequently, the q2-feature-classifier plugin was employed to assign taxonomy to representative sequences via a trained Naive Bayes classifier. Taxonomic classification was then performed using classify-sklearn, and the designated sequences were archived along with the trained classifier. To facilitate efficient association of taxonomy with the ASV table, taxon bar charts were generated and downloaded in CSV format from view.qiime2.org.

### Statistical analysis

Statistical analyses were performed in R v.3.5.3 except where specified^26^. All bar graphs and line diagrams were built using the ggplot2 package^27^. The normalization of read counts derived from 16S and ITS rRNA gene sequences was executed utilizing the DESeq2 package^28^. The alpha diversity metrics including Shannon (for diversity), Simpson (for proportional abundance), and Chao1 (for microbial richness) were computed using the diversity functions (specifically for Shannon and Simpson) and the estimateR function (for Chao1)^29^. Subsequently, these indices were graphically represented in a boxplot, facilitating the examination of spatial and temporal fluctuations inherent in these diversity metrics. To elucidate patterns of beta diversity within the microbial community, principal coordinate analysis (PCoA) was employed. This analysis serves as a valuable tool for elucidating the distinctions and similarities among communities. Distances were computed using the Bray–Curtis dissimilarity index, using the vegan package^30^ and vegdist() function. Permutational multivariate analysis of variance (PERMANOVA) was conducted to assess the statistical significance of observed differences, enabling the evaluation of temporal and spatial variability. These analyses were executed using the adonis2() function^31^. Statistically significant bacterial and fungal taxa differentiating the various groups were assessed. Subsequently, these findings were visually represented using a volcano plot^32^. Spearman correlation analysis was employed to assess the correlations among bacterial and fungal genera within the groups, in relation to anthropometric, biochemical, and dietary variables. This analysis was executed using the cor.test() function. A heatmap of Spearman’s rank correlation coefficients and *p*-values (0.05) was generated to visually represent the strength and significance of these associations. Additional statistical analyses such as Wilcoxon tests were conducted using wilcox.test() function^26^. To control for false positives from multiple tests, the Benjamini–Hochberg method with a 5% FDR threshold was applied using the 'p.adjust' function^33^. Linear discriminant analysis (LDA) was implemented using MASS (7.3–58.3)^30^.

### Ethics approval and consent to participate

This study was approved by The Instituto de Seguridad y Servicios Sociales de los Trabajadores del Estado (ISSSTE) Ethics Committee approved the study in Mexico (ISSSTE/CEI/2018/241), confirming that all experiments were performed in accordance with relevant guidelines and regulations.

## Results

In this study, we assessed the IBac (Intestinal Bacteriota) and IMy (Intestinal Mycobiota) profiles of 10 healthy-weight, 10 overweight, and 10 obese subjects, categorized according to the World Health Organization body mass index (BMI) classification. Anthropometric, dietary, and biochemical analyses were also conducted on each participant. The study cohort predominantly consisted of women, with an average age of 29.53 ± 7.85 years. Notably, significant differences in anthropometric parameters were observed among the healthy-weight, overweight, and obese groups. The percentage of fat mass increased progressively with BMI in all three groups (26.64 ± 9.79%, 29.01 ± 4.80%, and 37.40 ± 4.69%, respectively), whereas the percentage of lean mass exhibited a corresponding decrease (30.30 ± 0.09%, 24.9 ± 3.92%, and 23.52 ± 3.39%, respectively) (*p* < 0.0041). Waist average circumferences also escalated with BMI, with values of 77.23 ± 8.88 cm, 81.96 ± 4.36 cm, and 101.58 ± 13.12 cm in the healthy-weight, overweight, and obese groups, respectively (*p* < 0.0001) (Table 1). Biochemical analyses showed statistically significant distinctions among the groups. In particular, the obese group exhibited elevated levels of ALT, GGT enzymes, triglycerides, and glucose (*p* < 0.05), while HDL levels were higher in the healthy-weight group (54.04 ± 12.05) compared to the overweight (41.17 ± 6.33) and obese (38.08 ± 2.67) groups (*p* < 0.003). Regarding dietary parameters, kilocalories, carbohydrates, proteins, simple and complex carbohydrates, meat, and vegetable intake were notably higher in the obese group in comparison to the healthy-weight and overweight groups. However, only protein and meat consumption had significant statistical differences (*p* < 0.05). Significant differences remained after FDR correction (*p* < 0.05) (Table S1).

**Table 1:** Sociodemographic, anthropometric, biochemical, and dietary characteristics of the participants.

Characteristics	Groups of participants			*p* value
	Healthy-weight	Overweight	Obesity	
n	10	10	10	
Gender (female/male)	7/3	7/3	6/4	
Average age (years) (mean ± SD)	32 ± 7.65	29.90 ± 10.01	26.5 ± 4.62	0.2717
Average BMI (kg m^–2^)	22.87	26.49	35.7	0.0001*
Waist circumference (cm)	77.23 ± 8.88	81.96 ± 4.36	101.58 ± 13.12	0.0001*
Hip circumference (cm)	98.24 ± 4.58	102.55 ± 5.26	117.76 ± 15.59	0.0004*
Waist-hip ratio	0.79	0.79	0.86	0.0217*
Fat mass (%)	26.64 ± 9.79	29.01 ± 4.80	37.4 ± 4.69	0.0041*
Lean mass (%)	30.30 ± 9.09	24.9 ± 3.92	23.52 ± 3.39	0.0445*
Serum iron (μg/dL)	109.2 ± 36.41	93.98 ± 24.31	81.77 ± 21.99	0.1136
Total proteins (g/dL)	7.11 ± 0.32	7.12 ± 0.25	7.16 ± 0.24	0.9114
Total bilirubin (mg/dL)	0.69 ± 0.15	0.60 ± 0.28	0.86 ± 0.52	0.3169
ALT/GPT (U/L)	24.20 ± 15.79	25.64 ± 9.81	47.10 ± 27.84	0.0222*
AST/GOT (U/L)	36.05 ± 30.31	22.74 ± 6.00	60.22 ± 50.221	0.0606
GGT (U/L)	11.00 ± 7.64	19.20 ± 8.28	22.60 ± 9.16	0.0137*
Cholesterol (mg/dL)	169.60 ± 29.84	165.40 ± 22.90	170.80 ± 25.99	0.8915
HDL (mg/dL)	54.04 ± 12.05	41.17 ± 6.33	38.08 ± 2.67	0.0003*
Triglycerides (mg/dL)	93.30 ± 62.18	111.60 ± 27.16	188.10 ± 99.40	0.012*
Glucose (mg/dL)	79.20 ± 4.66	77.20 ± 3.04	85.30 ± 11.13	0.0465*
Kilocalories (kcal)	1760.9 ± 404.43	1636.50 ± 226.45	1844.40 ± 313.66	0.3644
Carbohydrates (kcal)	832.80 ± 277.88	796.00 ± 239.33	1004.40 ± 189.15	0.1326
Proteins (kcal)	360.20 ± 100.28	324.80 ± 78.98	386.80 ± 114.79	0.0001*
Lipids (kcal)	567.90 ± 235.39	515.70 ± 93.24	453.10 ± 146.03	0.3286
Simple carbohydrates (kcals)	28.00 ± 32.93	72.00 ± 90.03	88.00 ± 74.95	0.161
Complex carbohydrates (kcals)	804.80 ± 253.87	724.00 ± 244.62	944.20 ± 179.89	0.1117
Dairy products (kcals)	88.20 ± 87.54	64.80 ± 55.77	32.40 ± 30.00	0.2313
meat products (kcals)	159.60 ± 98.81	154.00 ± 68.90	212.80 ± 78.31	0.0001*
Vegetables (kcals)	112.40 ± 30.48	106.00 ± 55.40	139.60 ± 38.15	0.1928

Values represent the mean ± SD. *Values with statistically significant difference (analysis of variance, *p* < 0.05).

*BMI* Body Mass Index. *ALT/GPT* alanine transaminase. *AST/GOT* Aspartate aminotransferase. *GGT* gamma glutamyl transferase. *HDL* high density lipoprotein.

**Figure 1:**
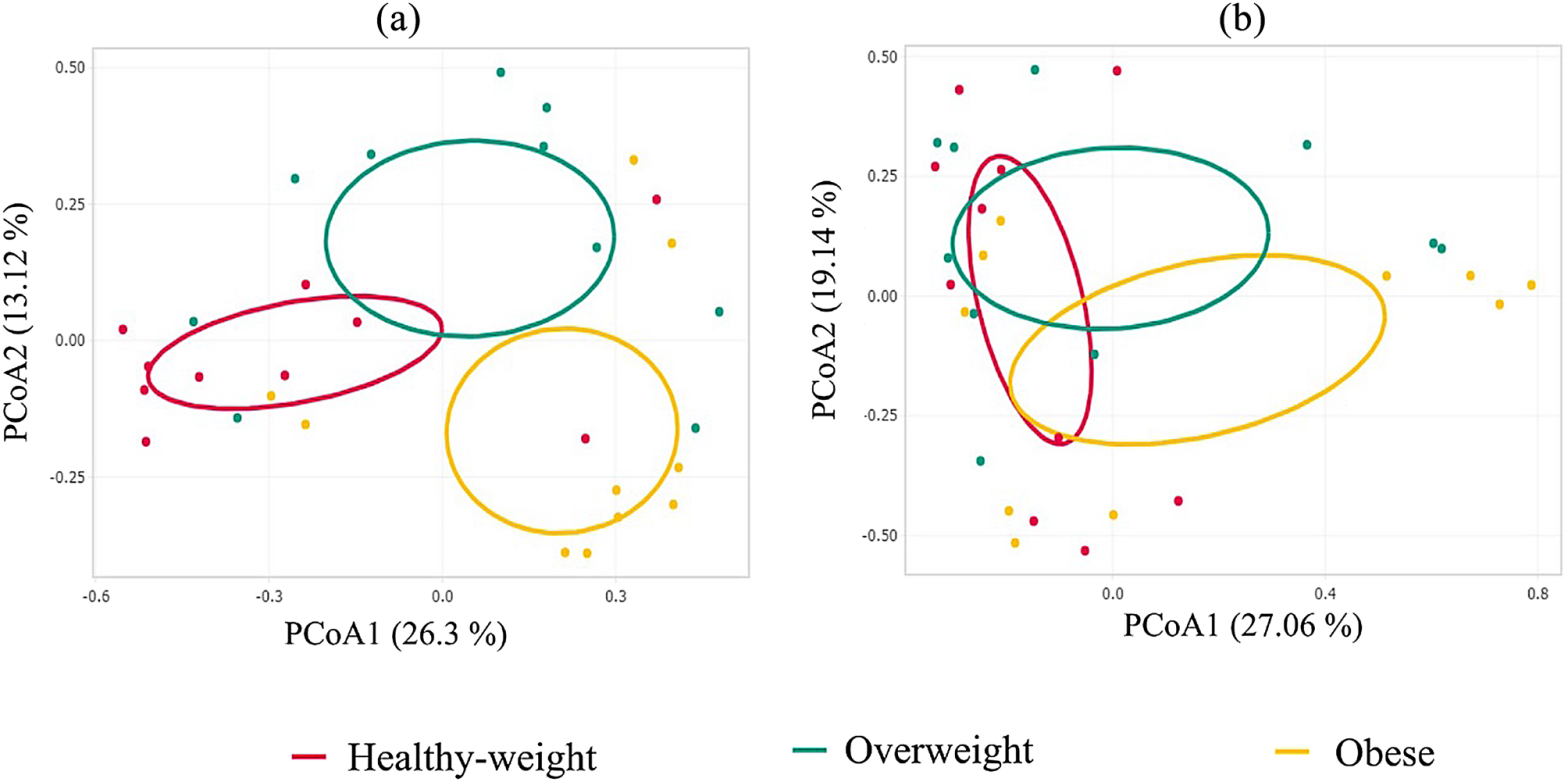
Beta diversity analysis of the dataset focusing on the bacterial (**a**) and fungal (**b**) genera present in the gut microbiota of the participants.

To elucidate the gut microbial composition, we sequenced samples using the 16S and ITS rRNA gene genetic markers to determine bacterial and fungal microorganisms, respectively. Sequencing data analysis revealed a total of 4,379,541 raw reads obtained for the V3–V4 hypervariable region of the 16S rRNA gene, while the ITS1 region yielded 3,775,592 reads. SILVA reference database analysis classified 74.70% of these reads as bacteria, while UNITE database analysis identified 32.36% as fungi (Table S2). The rarefaction curve illustrates the attainment of an asymptotic phase in the sampled data (Fig. S1). Interestingly, at the genus level, no significant differences were detected in the analysis of alpha diversity (including the Shannon index, Chao 1, and Simpson) for either IBac or IMy among the three study groups (Figs. S2, S3, respectively). Notably, bacteria displayed higher alpha diversity values compared to fungal communities across all three study groups. Healthy individuals exhibited lower alpha diversity in their bacterial communities compared to overweight and obese subjects. The Bray–Curtis dissimilarity metric was employed to perform beta diversity analysis (Fig. 1). The outcomes were graphically represented using Principal Coordinates Analysis (PCoA) at the genus level for both IBac and IMy. The PCoA plot for IBac indicated that the overweight group did not overlap with either the obesity or healthy-weight groups. This suggests statistically significant differences between the groups (*p* < 0.028) (Fig. 1a). In contrast, the PCoA plot for IMy showed overlapping clusters, revealing no statistically significant differences among the groups (*p* < 0.677) (Fig. 1b).

**Figure 2:**
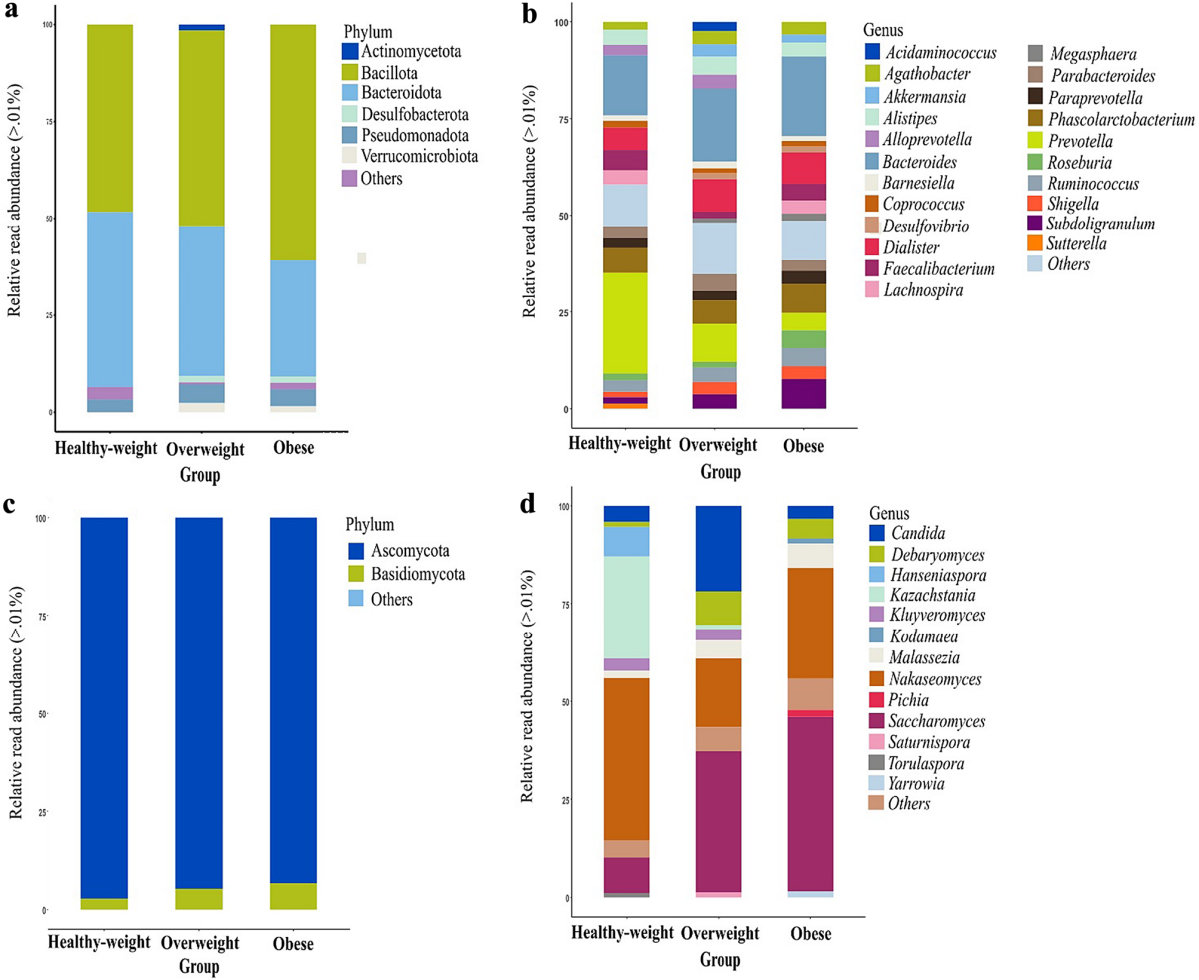
Taxonomic assignments of the intestinal bacteriota (IBac) and intestinal mycobiota (IMy) of participants, presented at the phylum (**a**, **c**) and genus (**b**, **d**) levels, respectively, using the QIIME 2.0 software.

The taxonomic distribution of bacterial and fungal communities at the phylum and genus levels is presented in Fig. 2. Bacillota and Bacteroidota were the predominant phyla across all three study groups (Fig. 2a). Bacillota exhibited an increasing trend with rising BMI in the healthy-weight, overweight, and obese groups (48.32%, 50.43%, and 60.84%, respectively), while Bacteroidota displayed the opposite pattern, decreasing with elevated BMI in these groups (45.17%, 38.79%, and 29.95%, respectively). Pseudomonadota (previously Proteobacteria^11^) accounted for 3.28%, 4.81%, and 4.36% of the microbial composition in healthy-weight, overweight, and obese subjects, respectively. At the genus level, *Prevotella* exhibited a higher prevalence in the healthy-weight group (26.05%), gradually declining in the overweight and obese groups (9.87% and 4.51%, respectively). *Bacteroides* showed substantial abundance in all three groups, comprising 15.52%, 18.88%, and 20.51% in healthy-weight, overweight, and obese groups, respectively (Fig. 2b and Table S3). Overweight and obese individuals had elevated abundance of *Akkermansia*, *Dialister*, *Phascolarctobacterium*, *Subdoligranulum*, and *Ruminococcus*, compared to the healthy-weight group.

In the analysis of IMy, Ascomycota and Basidiomycota were the dominant phyla across all three groups. Ascomycota constituted 97.19%, 94.72%, and 93.26% in the healthy-weight, overweight, and obese groups, respectively, while Basidiomycota accounted for 2.81%, 5.27%, and 6.74% in the healthy-weight, overweight, and obese groups, respectively (Fig. 2d). Among the fungal genera, *Nakaseomyces* exhibited the highest prevalence in all three groups, representing 41.39%, 17.48%, and 28.33% in the healthy-weight, obese, and overweight groups, respectively. *Saccharomyces* accounted for 9.15%, 36.00%, and 44.52% in the healthy-weight, overweight, and obese groups, respectively. *Candida* exhibited elevated abundance in overweight individuals (21.86%), followed by the healthy-weight group (4.15%) and the obese group (3.35%). *Debaryomyces* displayed higher prevalence in overweight and obese subjects (8.64% and 4.96%, respectively) compared to healthy-weight subjects (1.15%). *Kazachstania*, *Kluyveromyces*, and *Hanseniaspora* were notably more prevalent in the healthy-weight group (26.29%, 3.09%, and 7.52%, respectively) compared to the overweight (1.10%, 2.71%, and 0.5%, respectively), and obese groups (0.01%, 0.60%, and 0.01%, respectively) (Table S4). Additionally, *Pichia* was abundant in obese subjects (1.84%).

**Figure 3:**
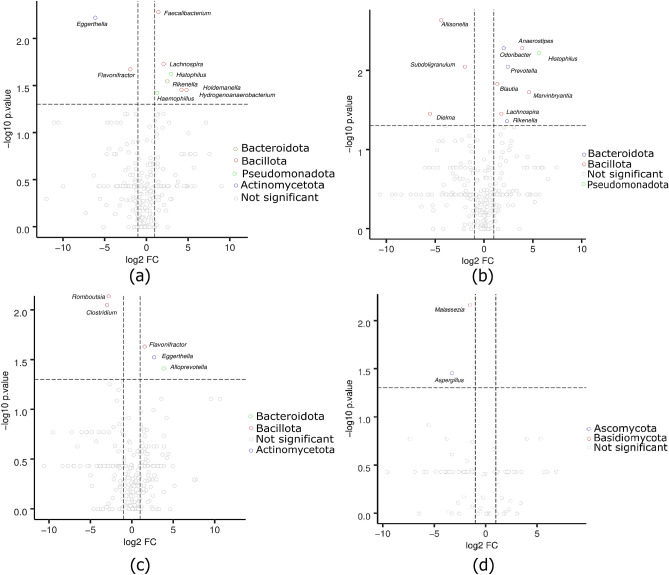
Volcano plots show statistically significant differences between weight groups in bacterial and fungal genus abundance. The x-axis shows the log2 of the fold change and the y-axis shows the -log of the *p*-value. (**a**) bacterial genera between the overweight and healthy-weight groups, (**b**) bacterial genera between the obese and healthy-weight groups, (**c**) bacterial genera between the obese and overweight groups, and (**d**) fungal genera between the obese and healthy-weight groups.

Volcano plots revealed distinct statistical differences in genus abundance in bacterial and fungal genera across weight groups (Fig. 3). Among bacterial genera, *Faecalibacterium*, *Lachnospira*, *Histophilus*, *Rikenella*, *Holdemanella*, *Hydrogenoanaerobacterium*, and *Haemophillus* were found to be significantly different between the healthy-weight and overweight groups (Fig. 3a). In comparison to obese subjects, healthy-weight subjects showed differential taxa including *Prevotella*, *Blautia*, *Lachnospira*, *Rikenella*, *Anaerostipes*, *Odoribacter*, *Marvinbryantia* and *Histophilus*, while obese subjects had three differential taxa, namely *Allisonella*, *Subdoligranulum* and *Dielma* (Fig. 3b). Additionally, *Lachnospira*, *Romboutsia* and *Clostridium* were found to be differential genera in obese subjects in comparison to the overweight group, which had *Flavonifractor*, *Eggerthella* and *Alloprevotella* as differential genera (Fig. 3c). In terms of fungal genera, *Malassezia* and *Aspergillus* were differential genera in obese subjects (Fig. 3d).

**Figure 4:**
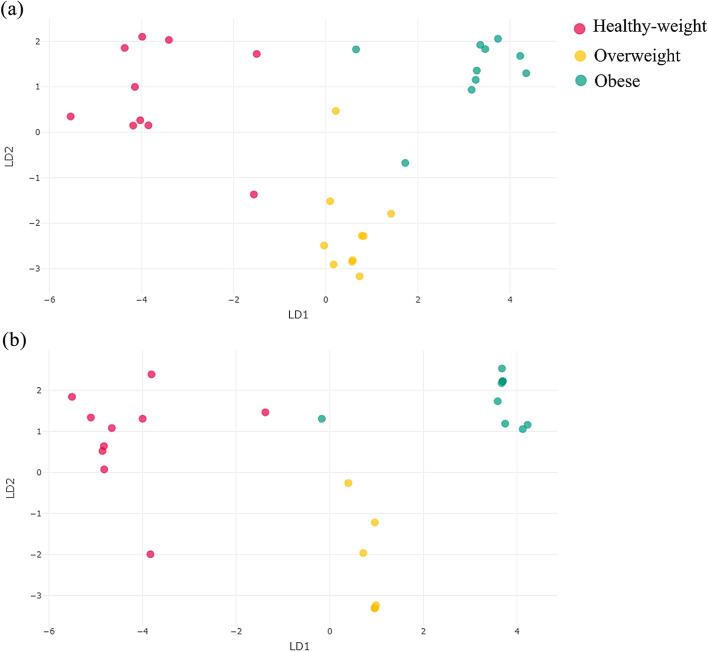
Comparison of the linear discriminant analysis of bacterial genera (**a**) and fungal genera (**b**) found in the intestinal microbiota of healthy-weight, overweight, and obese groups.

**Figure 5:**
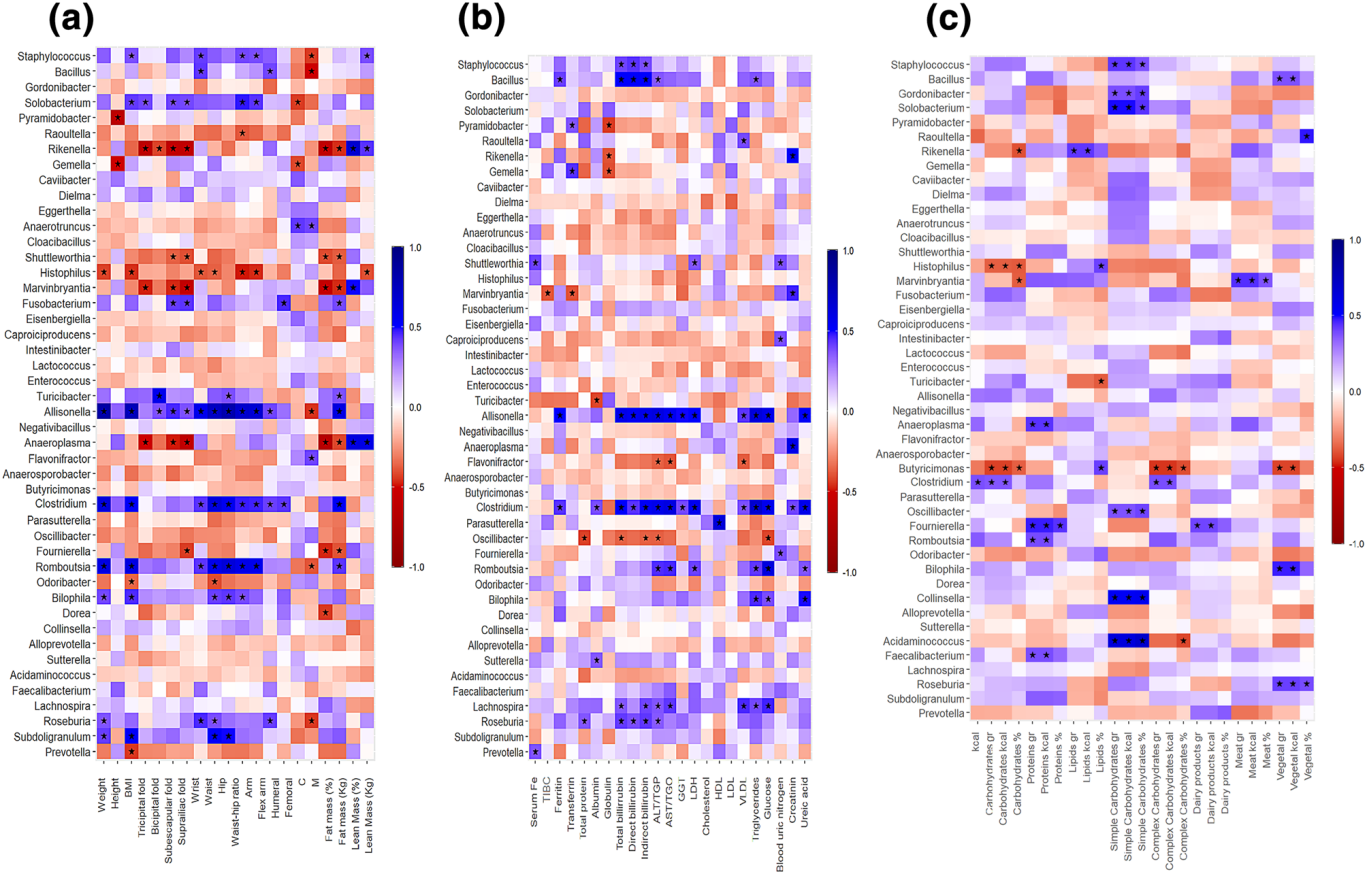
Spearman correlation analysis of intestinal bacterial genera among the healthy-weight, overweight, and obese groups with anthropometric (**a**), biochemical (**b**), and dietary (**c**) variables. Total iron-binding capacity (TIBC) high-density lipoprotein (HDL), low-density lipoprotein (LDL), very low-density lipoprotein (VLDL), alanine aminotransferase (ALT), aspartate aminotransferase (AST), glutamic gamma transferase (GGT), and lactate dehydrogenase (LDH). *p < 0.05.

To analyze the associations between bacterial and fungal genera and weight status, a linear discriminant analysis (LDA) utilizing multi-mode predictor variables was employed. The LDA classified samples into distinct groups corresponding to healthy-weight, overweight, and obese individuals (Fig. 4). The scatterplots in Fig. 4a and b show the coefficients of the linear discriminant functions, revealing a pattern for the data in the three groups with overlapping areas. This indicates that the discriminant functions can distinguish between the presence of bacterial and fungal genera in healthy-weight, overweight, and obese subjects. The correlation was observed among anthropometric (Fig. 5a), biochemical (Fig. 5b), and dietetic (Fig. 5c) parameters in relation to the bacterial genera identified in healthy-weight, overweight, and obese groups. The bacterial genera with high abundance in the healthy-weight group showed positive correlations with parameters related to good health and negative relationships with parameters related to weight gain. For example, *Histophilus* showed a positive correlation with high-density lipoproteins (HDL) and a negative correlation with weight gain, BMI, waist and hip circumferences, carbohydrate intake, LDL cholesterol, and triglycerides (*p* < 0.05). *Faecalibacterium* showed a positive correlation with HDL and kilocalories intake from protein, and a negative correlation with fat mass and low-density cholesterol (LDL) (*p* < 0.05). *Lachnospira* was positively correlated with triglycerides, glucose, and very low-density lipoprotein (VLDL) cholesterol (*p* < 0.05). It also showed a negative correlation with simple carbohydrates such as sucrose, but this correlation did not reach statistical significance. *Odoribacter* showed a positive correlation with HDL and a negative correlation with BMI, waist circumference, glucose, and triglycerides, as well as carbohydrates intake, simple carbohydrates intake, and kilocalories. *Prevotella* had a negative correlation with BMI and carbohydrates intake (*p* < 0.05). In contrast, the bacterial genera that exhibited high abundance in the overweight and obese groups showed a positive correlation with parameters associated with obesity. *Eggerthella* showed a positive correlation with LDL cholesterol, total cholesterol, and simple carbohydrates intake (*p* < 0.05). *Allisonella* showed a positive correlation with weight gain, BMI, biceps skinfold, subscapular skinfold, suprailiac skinfold, waist and hip circumferences, fat mass, VLDL cholesterol, triglycerides, serum glucose, carbohydrate intake, and kilocalories (*p* < 0.05). *Dielma* showed a positive correlation with weight, BMI, triceps skinfold, subscapular skinfold, suprailiac skinfold, fat mass, kilocalories, and carbohydrates intake. However, the correlation did not reach statistical significance. *Clostridium* was positively correlated with obesity-related parameters such as weight gain, waist and hip circumferences, BMI, fat mass, triglycerides, glucose, VLDL cholesterol, kilocalories, and carbohydrate intake (*p* < 0.05).

**Figure 6:**
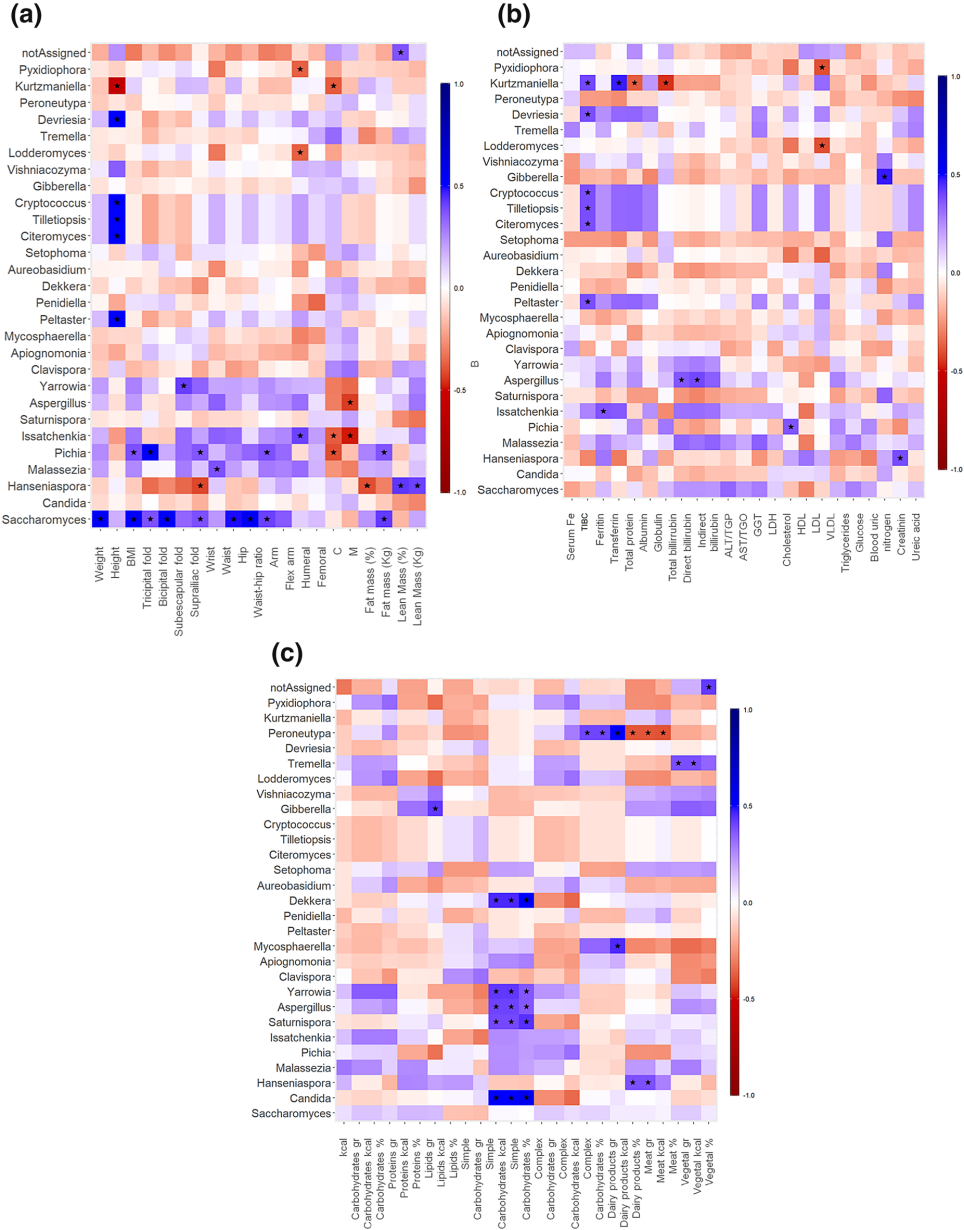
Spearman correlation analysis of intestinal fungal genera among the healthy-weight, overweight, and obese groups with anthropometric (**a**), biochemical (**b**), and dietary (**c**) variables. Total iron-binding capacity (TIBC) high-density lipoprotein (HDL), low-density lipoprotein (LDL), very low-density lipoprotein (VLDL), alanine aminotransferase (ALT), aspartate aminotransferase (AST), glutamic gamma transferase (GGT), and lactate dehydrogenase (LDH). *p < 0.05.

The correlation was observed among anthropometric (Fig. 6a), biochemical (Fig. 6b), and dietetic (Fig. 6c) parameters and the intestinal mycobiota in healthy-weight, overweight, and obese groups. *Aspergillus* and *Malassezia* showed a positive correlation with anthropometric parameters related to obesity, such as weight, BMI, subscapular and suprailiac skinfolds, waist, hip, and arm circumferences, but this correlation was not statistically significant. *Saccharomyces* exhibited a positive correlation with weight, BMI, triceps skinfold, biceps skinfold, and suprailiac skinfold, as well as with high fat mass (*p* < 0.05). *Pichia* showed a positive correlation with BMI, triceps skinfold, suprailiac skinfold, arm circumference, and fat mass (*p* < 0.05). *Yarrowia* displayed a positive correlation with weight, BMI, tricipital skinfold, suprailiac skinfold, and subscapular skinfold, waist and hip circumferences, and fat mass; however, it did not reach statistical significance except for the subscapular fold (*p* < 0.05). The correlation matrix of mycobiota and biochemical parameters showed fewer statistically significant correlations. *Aspergillus* showed a positive correlation with total bilirubin and direct bilirubin, whereas *Pichia* showed a positive correlation with total cholesterol (*p* < 0.05). *Saccharomyces* demonstrated a positive correlation with VLDL cholesterol, triglycerides, and glucose, and a negative correlation with total cholesterol, HDL, and LDL cholesterol, although this difference was not statistically significant. *Candida*, *Yarrowia*, *Aspergillus*, *Saturnispora* and *Dekkera* showed a positive correlation with the consumption of simple carbohydrates (*p* < 0.05). *Hanseniaspora* presented a positive correlation with meat consumption (*p* < 0.05). *Malassezia* and *Pichia* showed a positive correlation with kilocalories and carbohydrate consumption; however, this difference was not statistically significant.

## Discussion

Prior to this study, the cultivable intestinal mycobiota had been evaluated using culture-dependent methods in combination with the MALDI TOF technique, as reported by García-Gamboa et al.^15^. In this study, we present the results of an analysis of the intestinal bacteriota and mycobiota using molecular techniques, allowing for a comparison with the intestinal mycobiota examined in the previous study. This study identified the IBac and IMy in healthy-weight, overweight, and obese Mexican subjects, and related these findings to anthropometric, biochemical, and dietary parameters. While bacteria are the predominant microorganisms found in the gut microbiome, fungal microorganisms also play a crucial role, representing approximately 0.03–2.0% of the total microorganisms present^15^. Despite their lower abundance, it is important to note that fungal cells can be up to 100 times larger than prokaryotic cells, making the gut mycobiome an important biomass at the intestinal level. Moreover, research has shown that the gut mycobiome can affect the host metabolism, making it an interesting component to study in different human physiological states^34^. Alpha diversity of gut bacteria and fungi showed no significant differences across healthy-weight, overweight, and obese groups, consistent with^35^, where the authors reported that changes in alpha diversity were not correlated with obesity. Lower alpha diversity was observed in bacterial communities of healthy individuals relative to those in overweight/obese counterparts. Potential contributing factors include the abundance of specific taxa like *Prevotella*, as captured by indices such as Shannon and Simpson^36^. In another study, Kim et al.^37^ found less bacterial diversity in obese subjects with metabolic disease compared to obese people with healthy metabolism. While previous research connects bacterial diversity to metabolic processes^38^, suggesting limited understanding of its role. Some studies have reported alterations in fungal diversity. For instance, Iliev and Cadwell (2021)^39^ highlighted changes in fungal diversity in intestinal diseases such as Crohn’s disease. Though fungal diversity may link to specific pathologies, its role in disease development needs further research. Other studies have shown that bacterial diversity is higher than fungal diversity in the gut^40,41^, which is consistent with our findings. The disparity in diversity patterns observed between bacterial and fungal communities across metabolic states might be attributable to the limited sample size employed in this study and the inherently higher interindividual variability of the gut mycobiome compared to the bacterial community^42^. Further studies with larger sample size are needed to discover the precise characteristics of the gut mycobiome dynamics in individuals with obesity. It has been reported that obese individuals have a higher abundance of Bacillota and a lower abundance of Bacteroidota, with the Bacillota/Bacteroidota ratio frequently cited as a hallmark of obesity^12^. Overweight and obese subjects in our study showed a higher Bacillota/Bacteroidota ratio than healthy ones. Diets linked to obesity, like high-carb/high-fat^43^, increase this ratio and disturb gut balance (dysbiosis)^44^. Intestinal bacteria from Bacillota and Bacteroidota produce enzymes (CAZymes) that digest complex dietary sugars, potentially contributing to the host's energy intake^45^. Conversely, calorie-restricted diets and weight loss increase Bacteroidota^12^, linked to reduced fat mass. Walker & Hoyles (2023)^46^ highlight the common use of the Bacillota/Bacteroidota ratio in obesity research. However, they warn of oversimplification at higher taxonomic levels due to internal variability within phyla. Additionally, they suggest moving beyond relative abundance measures like DNA sequencing to more robust techniques like qPCR for accurate analysis. The *Bacteroides*/*Prevotella* ratio has also been proposed as a possible biomarker for identifying nutritional states and managing obesity^47^. *Bacteroides*, linked to high-fat diets, dominated in overweight and obese groups. Higher *B. vulgatus* in healthy individuals suggests a protective role against obesity and atherosclerosis^48^. This is consistent with previous research that has linked *Bacteroides* to high-fat diets and Western-style diets, as the groups with the highest BMI in this study also showed high fat intake^15^. *Prevotella*, associated with fiber intake, was more abundant in healthy-weight participants. *P. copri's* increased presence in healthy subjects may improve glucose metabolism^49^. In this study, groups with high BMI had a higher presence of *Akkermansia*, specifically *Akkermansia muciniphila*. However, these results differ from those reported in previous studies by Roshanravan et al.^50^ and Xu et al.^51^, who found an inverse relationship between *A. muciniphila* and weight gain. Other studies have reported that the presence of *A. muciniphila* in obese subjects can improve certain metabolic parameters, such as insulin sensitivity, total plasma cholesterol, glucose, and triglycerides, leading to an overall improvement in metabolic state^52,53^. Our findings are consistent with these studies, as overweight and obese participants with a high abundance of *A. muciniphila* exhibited a healthier lipid and glucose profile compared to those with a lower abundance of this microorganism. In this study, Ascomycota and Basidiomycota dominated intestinal mycobiota across all groups, similar to other studies^15^. Key fungal genera found included *Nakaseomyces*, *Saccharomyces*, *Kazachstania*, *Candida*, *Hanseniaspora*, and *Malassezia*, mirroring prior findings suggesting *Saccharomyces* as the most abundant gut fungus, followed by *Malassezia* and *Candida*^41^. Musumeci et al.^54^ reported that *Candida albicans* is an opportunistic pathogen and a major component of the intestinal mycobiome. They also described the relationship between the intestinal mycobiota and diet, showing a positive correlation between *Candida* and carbohydrate consumption. Our study found similar results, as the overweight and obese groups reported higher carbohydrate intake. This could be related to *Candida*'s ability to break down polysaccharides in food, releasing simple sugars that can be fermented by intestinal bacteria such as *Ruminococcus*^55^ or *Prevotella*^56^. Bardagjy and Steinberg (2019)^57^ reported that *C. albicans* has a positive correlation with obesity-related parameters and metabolic diseases, showing a negative correlation with high-density lipoproteins and lean mass. Our previous study of the cultivable mycobiota in the gut found a high presence of *C. albicans*, particularly in the obese group^15^. In the current study, the overweight group showed a higher prevalence of *Candida*, with *C. zeylanoides* being the most abundant species. While reports indicate that *C. zeylanoides* can inhabit the gastrointestinal tract^58^, its role in host physiology and pathology is not well studied. In addition, *C. parapsilosis* was highly abundant in overweight subjects. This yeast has been linked to high-fat diets and the development of obesity, as it can secrete lipases that break down and utilize fats in the gastrointestinal tract^59^. These findings are consistent with our previous study, where *C. parapsilosis* showed greater abundance in the overweight group^15^. The cultivable mycobiota overlooked important fungi like *Saccharomyces*, *Nakaseomyces*, or *Malassezia*, likely due to their unique growth requirements. *Malassezia*, for instance, needs stricter conditions than readily available media like Sabouraud Dextrose^41^. Overweight and obese individuals had slightly more Basidiomycota, possibly due to higher *Malassezia* levels. This microorganism, mainly found on skin^60^, is controversially linked to gut issues but was also present in healthy individuals in other studies^36,55^. Our study found *M. globosa* highest in overweight and obese, but its connection to obesity is unclear. *Malassezia* differed notably in obese individuals, but their link to weight gain is unclear. A positive correlation between *Saccharomyces* and BMI was observed, though its role in obesity development remains unknown^58^. However, *Saccharomyces boulardii* may reduce weight and BMI^61^, and its variations have been linked to weight changes^62^.

An important finding from a previous study of cultivable mycobiota was the high abundance of *Rhodotorula* in the obese group^15^. However, in the present study, *Rhodotorula* was not found in any of the groups. High abundance of *Rhodotorula* has been linked to intestinal dysbiosis^63^ and obesity, as it can produce saturated and unsaturated fatty acids by using glucose, acetic acid, and propionic acid as a carbon source^64^. Thus, it is possible to hypothesize that the intestinal mycobiota, particularly *Rhodotorula*, can modulate the production of fatty acids, energy, and nutritional status of the host, contributing to the development of obesity. The presence of *Kazachstania* in the healthy-weight group was similar to that found in the cultivable mycobiota study^15^. *Kazachstania exigua* was the main species found in both studies. While no studies have reported on the role of *K. exigua* in the human intestinal microbiota, some studies have noted its presence in the intestinal microbiota of animals, especially pigs^65^. This highlights the importance of *Kazachstania* in developing a healthy porcine microbiome, as it promotes the growth of bacteria that produce short-chain fatty acids^66^, which can be beneficial for intestinal health. Therefore, future studies should investigate the functions of this microorganism and its role in the human intestinal mycobiota. Witherden et al.^67^, reported an antagonistic effect between *Pichia* and *Candida*, meaning that an increase in the abundance of *Pichia* is associated with a reduction in *Candida*. This is consistent with the results obtained in the current study, where the obese group showed higher presence of *Pichia* than of *Candida albicans* compared to overweight and healthy-weight participants. The presence of *Pichia* in the obese group was also similar to the data reported by Rodríguez et al.^16^ and Gouba et al.^68^. The antagonistic effect of *Pichia* may be due to *Pichia* limiting nutrients and modulating the synthesis of growth and virulence factors against *Candida,* as reported by Mukherjee et al.^69^. This highlights the importance of investigating further the role of interactions between microorganisms that are part of the intestinal bacteriota and mycobiota, particularly in the development of overweight and obesity. An important finding revealed by both the current study and the cultivable mycobiota study^15^ was the distinct clustering of the healthy-weight, overweight, and obese groups based on the fungal microorganism abundance in the linear discriminant analysis. Although no reports have grouped individuals based on their BMI and fungal community composition, these results underscore the significance of further investigating the role of the intestinal mycobiota in obesity. *Candida* was found in overweight individuals, *C. parapsilosis* in healthy-weight and overweight groups, *Pichia* in the obese group, and *Kazachstania* in healthy-weight subjects in both studies. These shared findings through cultivable and sequencing techniques may generate interest in exploring the functional and microbial diversity aspects of the intestinal mycobiota, which may be approached from various perspectives such as human nutrition, health, disease, drug metabolism, and more.

## Conclusions

In conclusion, the gut microbiome in all three groups showed greater diversity in bacteria than fungal microorganisms. In the beta analysis at the genus level, a distinct cluster of bacteria was observed within the overweight group. The Bacillota/Bacteroidota and *Bacteroides*/*Prevotella* ratios positively correlated with BMI, which is an obesity-related parameter. Overweight and obese participants with higher abundance of *Akkermansia muciniphila* demonstrated a healthier lipid and glucose profile, in comparison to those with a lower abundance of *A. muciniphila*. The healthy-weight group showed differential bacterial genera, including *Faecalibacterium*, *Histophilus*, *Rikenella*, *Odoribacter*, and *Marvinbryantia*. These genera positively correlated with anthropometric, biochemical, and dietary parameters related to good health and ideal weight. Ascomycota and Basidiomycota were the most abundant phyla in the intestinal mycobiota of the three study groups, with Ascomycota showing higher abundance. *Nakaseomyces* and *Saccharomyce*s were the most abundant fungal genera across all three groups. In the healthy-weight group, *Nakaseomyces*, *Kazachstania*, *Kluyveromyces*, and *Hanseniaspora* were the most abundant genera, and they showed a negative relationship with BMI. Conversely, *Saccharomyces*, *Debaryomyces*, and *Pichia* were the most abundant genera in the overweight and obese groups and showed a positive relationship with BMI. *Candida* was most abundant in overweight subjects and showed a positive correlation with the consumption of simple carbohydrates. *Malassezia* and *Aspergillus* were the differential fungal genera in the obese group and were associated with anthropometric, biochemical, and dietary parameters related to weight gain.

### Supplementary Information

Supplementary Information.

## Data Availability

The data presented in this study are available on request from the corresponding author. The sequencing datasets used in this study are publicly available in the NCBI Sequence Read Archive (SRA) under the BioProject IDs: PRJNA1031701 and PRJNA1032058.

## Abbreviations

IBact: Intestinal bacteriota
IMy: Intestinal mycobiota
BMI: Body mass index
TIBC: Total iron-binding capacity
HDL: High-density lipoprotein
LDL: Low-density lipoprotein
VLDL: Very low-density lipoprotein
ALT: Alanine aminotransferase
AST: Aspartate aminotransferase
GGT: Glutamic gamma transferase
LDH: Lactate dehydrogenase
ITS: Internal transcribed spacer
DNA: Deoxyribonucleic acid
RNA: Ribonucleic acid
qPCR: Quantitative polymerase chain reaction
ASV: Amplicon sequence variants
LDA: Linear discriminant analysis

## References

[1] Gebrayel P, Nicco C, Al Khodor S, Bilinski J, Caselli E, Comelli EM (2022). Microbiota medicine: Towards clinical revolution. J. Transl. Med..

[2] Retnakumar RJ, Nath AN, Nair GB, Chattopadhyay S (2022). Gastrointestinal microbiome in the context of *Helicobacter pylori* infection in stomach and gastroduodenal diseases. Prog. Mol. Biol. Transl. Sci..

[3] Matijašić M, Meštrović T, Čipčić Paljetak H, Perić M, Barešić A, Verbanac D (2020). Gut microbiota beyond bacteria—mycobiome, virome, archaeome, and eukaryotic parasites in IBD. Int. J. Mol. Sci..

[4] Xiao H, Kang S (2020). The role of the gut microbiome in energy balance with a focus on the gut-adipose tissue axis. Front. Genet..

[5] Leigh S-J, Morris MJ (2020). Diet, inflammation and the gut microbiome: Mechanisms for obesity-associated cognitive impairment. Biochim. Biophys. Acta BBA Mol. Basis Dis..

[6] Kwok S, Adam S, Ho JH, Iqbal Z, Turkington P, Razvi S (2020). Obesity: A critical risk factor in the COVID-19 pandemic. Clin. Obes..

[7] Lacroix M, Riscal R, Arena G, Linares LK, Le Cam L (2020). Metabolic functions of the tumor suppressor p53: Implications in normal physiology, metabolic disorders, and cancer. Mol. Metab..

[8] Sehgal K, Khanna S (2021). Gut microbiota: A target for intervention in obesity. Expert Rev. Gastroenterol. Hepatol..

[9] Halfvarson J, Brislawn CJ, Lamendella R, Vázquez-Baeza Y, Walters WA, Bramer LM (2017). Dynamics of the human gut microbiome in inflammatory bowel disease. Nat. Microbiol..

[10] Yun L, Li W, Wu T, Zhang M (2022). Effect of sea cucumber peptides on the immune response and gut microbiota composition in ovalbumin-induced allergic mice. Food Funct..

[11] Oren A, Garrity GM (2021). Valid publication of the names of forty-two phyla of prokaryotes. Int. J. Syst. Evol. Microbiol..

[12] Magne F, Gotteland M, Gauthier L, Zazueta A, Pesoa S, Navarrete P (2020). The firmicutes/bacteroidetes ratio: A relevant marker of gut dysbiosis in obese patients?. Nutrients.

[13] Turnbaugh PJ, Hamady M, Yatsunenko T, Cantarel BL, Duncan A, Ley RE (2009). A core gut microbiome in obese and lean twins. Nature.

[14] Turnbaugh PJ, Ley RE, Mahowald MA, Magrini V, Mardis ER, Gordon JI (2006). An obesity-associated gut microbiome with increased capacity for energy harvest. Nature.

[15] García-Gamboa R, Kirchmayr MR, Gradilla-Hernández MS, Pérez-Brocal V, Moya A, González-Avila M (2021). The intestinal mycobiota and its relationship with overweight, obesity and nutritional aspects. J. Hum. Nutr. Diet..

[16] Mar Rodríguez M, Pérez D, Javier Chaves F, Esteve E, Marin-Garcia P, Xifra G (2015). Obesity changes the human gut mycobiome. Sci. Rep..

[17] Pérez JC (2021). Fungi of the human gut microbiota: Roles and significance. Int. J. Med. Microbiol..

[18] Kuziel GA, Rakoff-Nahoum S (2022). The gut microbiome. Curr. Biol..

[19] Marfell-Jones, M. J., Stewart, A. D. & de Ridder, J. H. International standards for anthropometric assessment. [Internet]. 2012 [cited 2023 Feb 4]. Available from: https://repository.openpolytechnic.ac.nz/handle/11072/1510

[20] Klindworth A, Pruesse E, Schweer T, Peplies J, Quast C, Horn M (2013). Evaluation of general 16S ribosomal RNA gene PCR primers for classical and next-generation sequencing-based diversity studies. Nucleic Acids Res..

[21] Gao B, Chi L, Zhu Y, Shi X, Tu P, Li B (2021). An introduction to next generation sequencing bioinformatic analysis in gut microbiome studies. Biomolecules.

[22] Bolyen E, Rideout JR, Dillon MR, Bokulich NA, Abnet CC, Al-Ghalith GA (2019). Reproducible, interactive, scalable and extensible microbiome data science using QIIME 2. Nat. Biotechnol..

[23] Callahan BJ, McMurdie PJ, Rosen MJ, Han AW, Johnson AJA, Holmes SP (2016). DADA2: High-resolution sample inference from Illumina amplicon data. Nat. Methods.

[24] Quast C, Pruesse E, Yilmaz P, Gerken J, Schweer T, Yarza P (2013). The SILVA ribosomal RNA gene database project: Improved data processing and web-based tools. Nucleic Acids Res..

[25] Kõljalg U, Nilsson RH, Abarenkov K, Tedersoo L, Taylor AFS, Bahram M (2013). Towards a unified paradigm for sequence-based identification of fungi. Mol. Ecol..

[26] Team RC. R: A language and environment for statistical computing. R Foundation for Statistical Computing, Vienna. R-Proj Org. (2013)

[27] Villanueva RAM, Chen ZJ (2019). ggplot2: Elegant graphics for data analysis (2nd edn.). Meas. Interdiscip. Res. Perspect..

[28] Love MI, Huber W, Anders S (2014). Moderated estimation of fold change and dispersion for RNA-seq data with DESeq2. Genome Biol..

[29] Díaz-Torres O, de Anda J, Lugo-Melchor OY, Pacheco A, Orozco-Nunnelly DA, Shear H (2021). Rapid changes in the phytoplankton community of a subtropical, shallow, hypereutrophic lake during the rainy season. Front. Microbiol..

[30] Oksanen, J., Simpson, G. L., Blanchet, F. G., Kindt, R., Legendre, P., Minchin, P. R. *et al*. Vegan: Community ecology package [Internet] (2022). [cited 2024 Jan 3]. Available from: https://cran.r-project.org/web/packages/vegan/index.html

[31] Warton DI, Wright ST, Wang Y (2012). Distance-based multivariate analyses confound location and dispersion effects. Methods Ecol. Evol..

[32] Dan X, Mushi Z, Baili W, Han L, Enqi W, Huanhu Z (2019). Differential analysis of hypertension-associated intestinal microbiota. Int. J. Med. Sci..

[33] Pacini C, Dempster JM, Boyle I, Gonçalves E, Najgebauer H, Karakoc E (2021). Integrated cross-study datasets of genetic dependencies in cancer. Nat. Commun..

[34] Fiers WD, Leonardi I, Iliev ID (2020). From birth and throughout life: Fungal microbiota in nutrition and metabolic health. Annu. Rev. Nutr..

[35] Castaner O, Goday A, Park Y-M, Lee S-H, Magkos F, Shiow S-ATE (2018). The gut microbiome profile in obesity: A systematic review. Int. J. Endocrinol..

[36] Herrera AM, Riera R, Rodríguez RA (2023). Alpha species diversity measured by Shannon’s H-index: Some misunderstandings and underexplored traits, and its key role in exploring the trophodynamic stability of dynamic multiscapes. Ecol. Indic..

[37] Kim M-H, Yun KE, Kim J, Park E, Chang Y, Ryu S (2020). Gut microbiota and metabolic health among overweight and obese individuals. Sci. Rep..

[38] Matey-Hernandez ML, Williams FMK, Potter T, Valdes AM, Spector TD, Menni C (2018). Genetic and microbiome influence on lipid metabolism and dyslipidemia. Physiol. Genom..

[39] Iliev ID, Cadwell K (2021). Effects of intestinal fungi and viruses on immune responses and inflammatory bowel diseases. Gastroenterology.

[40] Hallen-Adams HE, Suhr MJ (2017). Fungi in the healthy human gastrointestinal tract. Virulence.

[41] Nash AK, Auchtung TA, Wong MC, Smith DP, Gesell JR, Ross MC (2017). The gut mycobiome of the Human Microbiome Project healthy cohort. Microbiome.

[42] Shah S, Locca A, Dorsett Y, Cantoni C, Ghezzi L, Lin Q (2021). Alterations of the gut mycobiome in patients with MS. EBioMedicine.

[43] Won S-M, Chen S, Lee SY, Lee KE, Park KW, Yoon J-H (2020). *Lactobacillus sakei* ADM14 induces anti-obesity effects and changes in gut microbiome in high-fat diet-induced obese mice. Nutrients.

[44] Grigor’eva IN (2021). Gallstone disease, obesity and the firmicutes/bacteroidetes ratio as a possible biomarker of gut dysbiosis. J. Pers. Med..

[45] Kaoutari AE, Armougom F, Gordon JI, Raoult D, Henrissat B (2013). The abundance and variety of carbohydrate-active enzymes in the human gut microbiota. Nat. Rev. Microbiol..

[46] Walker AW, Hoyles L (2023). Human microbiome myths and misconceptions. Nat. Microbiol..

[47] Ortega-Santos CP, Whisner CM (2019). The key to successful weight loss on a high-fiber diet may be in gut microbiome *Prevotella* abundance. J. Nutr..

[48] Yoshida N, Yamashita T, Osone T, Hosooka T, Shinohara M, Kitahama S (2021). *Bacteroides* spp. promotes branched-chain amino acid catabolism in brown fat and inhibits obesity. iScience..

[49] Makki K, Deehan EC, Walter J, Bäckhed F (2018). The impact of dietary fiber on gut microbiota in host health and disease. Cell Host Microbe.

[50] Roshanravan, N., Bastani, S., Tutunchi, H., Kafil, B., Nikpayam, O., Mesri Alamdari, N. *et al*. A comprehensive systematic review of the effectiveness of *Akkermansia muciniphila*, a member of the gut microbiome, for the management of obesity and associated metabolic disorders. *Arch. Physiol. Biochem.*, 1–11 (2021). 10.1080/13813455.2021.1871760 33449810

[51] Xu Y, Wang N, Tan H-Y, Li S, Zhang C, Feng Y (2020). Function of *Akkermansia muciniphila* in obesity: Interactions with lipid metabolism, immune response and gut systems. Front. Microbiol..

[52] Dao MC, Everard A, Aron-Wisnewsky J, Sokolovska N, Prifti E, Verger EO (2016). *Akkermansia muciniphila* and improved metabolic health during a dietary intervention in obesity: Relationship with gut microbiome richness and ecology. Gut.

[53] Depommier C, Everard A, Druart C, Plovier H, Van Hul M, Vieira-Silva S (2019). Supplementation with *Akkermansia muciniphila* in overweight and obese human volunteers: A proof-of-concept exploratory study. Nat. Med..

[54] Musumeci S, Coen M, Leidi A, Schrenzel J (2022). The human gut mycobiome and the specific role of *Candida albicans*: Where do we stand, as clinicians?. Clin. Microbiol. Infect..

[55] Borgo F, Verduci E, Riva A, Lassandro C, Riva E, Morace G (2017). Relative abundance in bacterial and fungal gut microbes in obese children: A case control study. Child. Obes..

[56] Hills RD, Pontefract BA, Mishcon HR, Black CA, Sutton SC, Theberge CR (2019). Gut microbiome: Profound implications for diet and disease. Nutrients.

[57] Bardagjy AS, Steinberg FM (2019). Relationship between HDL functional characteristics and cardiovascular health and potential impact of dietary patterns: A narrative review. Nutrients.

[58] Raimondi S, Amaretti A, Gozzoli C, Simone M, Righini L, Candeliere F (2019). Longitudinal survey of fungi in the human gut: ITS profiling, phenotyping, and colonization. Front. Microbiol..

[59] Sun S, Sun L, Wang K, Qiao S, Zhao X, Hu X (2021). The gut commensal fungus, *Candida parapsilosis*, promotes high fat-diet induced obesity in mice. Commun. Biol..

[60] Spatz M, Richard ML (2020). Overview of the potential role of *Malassezia* in gut health and disease. Front. Cell. Infect. Microbiol..

[61] Stojanov S, Berlec A, Štrukelj B (2020). The influence of probiotics on the firmicutes/bacteroidetes ratio in the treatment of obesity and inflammatory bowel disease. Microorganisms.

[62] Mims TS, Abdallah QA, Stewart JD, Watts SP, White CT, Rousselle TV (2021). The gut mycobiome of healthy mice is shaped by the environment and correlates with metabolic outcomes in response to diet. Commun. Biol..

[63] Hof H (2019). Rhodotorula spp. in the gut—foe or friend?. GMS Infect. Dis..

[64] Kolouchová I, Schreiberová O, Sigler K, Masák J, Řezanka T (2015). Biotransformation of volatile fatty acids by oleaginous and non-oleaginous yeast species. FEMS Yeast Res..

[65] Bendová B, Piálek J, Ďureje Ľ, Schmiedová L, Čížková D, Martin J-F (2020). How being synanthropic affects the gut bacteriome and mycobiome: Comparison of two mouse species with contrasting ecologies. BMC Microbiol..

[66] Urubschurov V, Büsing K, Freyer G, Herlemann DPR, Souffrant W-B, Zeyner A (2017). New insights into the role of the porcine intestinal yeast, *Kazachstania slooffiae*, in intestinal environment of weaned piglets. FEMS Microbiol. Ecol..

[67] Witherden EA, Shoaie S, Hall RA, Moyes DL (2017). The human mucosal mycobiome and fungal community interactions. J. Fungi.

[68] Gouba N, Hien YE, Guissou ML, Fonkou MDM, Traoré Y, Tarnagda Z (2019). Digestive tract mycobiota and microbiota and the effects on the immune system. Hum. Microbiome J..

[69] Mukherjee PK, Sendid B, Hoarau G, Colombel J-F, Poulain D, Ghannoum MA (2015). Mycobiota in gastrointestinal diseases. Nat. Rev. Gastroenterol. Hepatol..

