# Supplementary material for: Associations between bacterial and fungal communities in the human gut microbiota and their implications for nutritional status and body weigh﻿t
Source: Sci Rep. 2024 Mar 8;14:5703. doi: 10.1038/s41598-024-54782-7 (PMC10923939; doi:10.1038/s41598-024-54782-7)
Supplement: Supplementary file 1 — Supplementary Information. [file 41598_2024_54782_MOESM1_ESM.docx]

**Associations between bacterial and fungal communities in the human gut microbiota and their implications for nutritional status and body weight**

Ricardo García-Gamboa ^1,2^, Carolina Senés-Guerrero ^3^, Osiris Díaz-Torres ^3,4^, Misael S. Gradilla-Hernández ^3,4^, Andrés Moya^5,6,7^, Vicente Pérez-Brocal ^5,6^, Alejandro Garcia-Gonzalez^2^, and Marisela González-Avila ^1*^

**Supplementary information**

Additional file 1:

Figure S1

Rarefaction curve using 16S rRNA gene sequences to analyze bacteria and ITS1 gene sequences for fungi across the healthy-weight, overweight, and obese cohorts. The range spans from 0 to 120,000,000.

Figure S2

Analysis of bacterial alpha diversity in the intestinal microbiota of healthy-weight, overweight, and obese cohorts. The Shannon (A), Chao 1 (B), and Simpson (C) index values were computed based on 16S rRNA gene samples at the genus level to assess microbial diversity.

Figure S3

Analysis of bacterial alpha diversity in the intestinal microbiota of healthy-weight, overweight, and obese cohorts. The Shannon (A), Chao 1 (B), and Simpson (C) index values were computed based on 16S rRNA gene samples at the genus level to assess microbial diversity.

Table S1

Anthropometric, biochemical, and dietary data with FDR-corrected *p*-values (adjusted *p* values) < 0.05 were considered significant.

Table S2

Number of reads obtained from sequencing data analysis.

Table S3

Read relative abundance of bacterial microorganisms at the phylum, genus, and species levels in the intestinal microbiota of the healthy-weight, overweight, and obese groups.

Table S4

Read relative abundance of fungal microorganisms at the phylum, genus, and species levels in the intestinal microbiota of the healthy-weight, overweight, and obese groups.

Figure S1


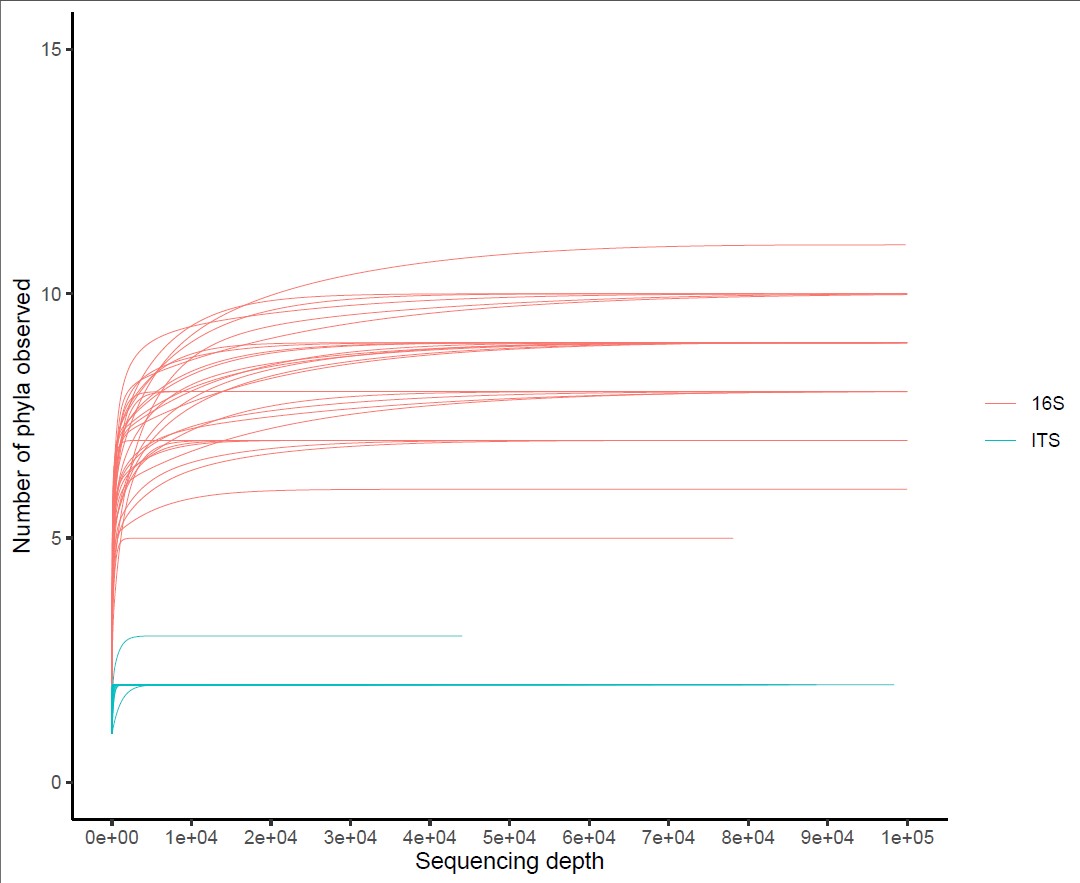


Figure S2


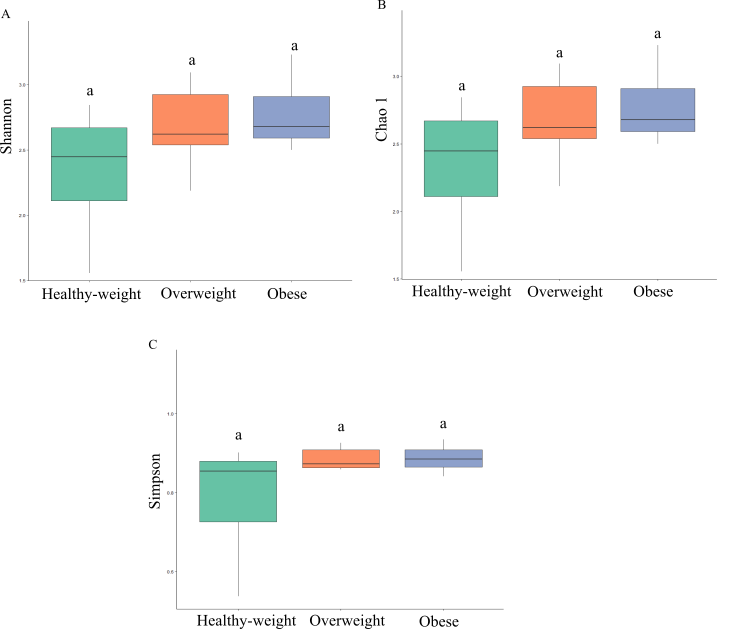


Figure S3


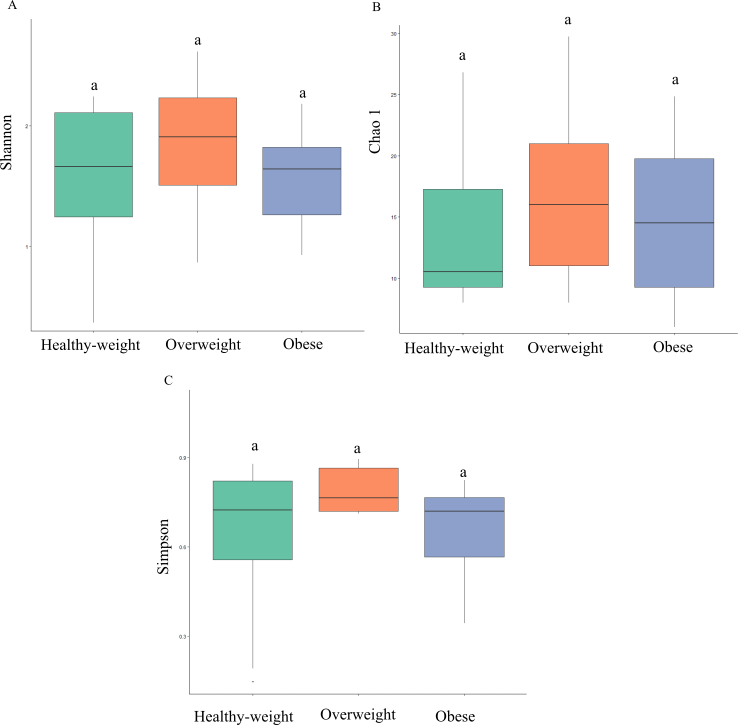


Table S1

**Supplementary Table 1.** Anthropometric, biochemical, and dietary data with FDR-corrected *p*-values (adjusted *p* values) < 0.05 were considered significant.

|  |  |  |
| --- | --- | --- |
| Variable | Valor_p | adjusted_p_values |
| BMI | 8.60165E-08 | 2.15041E-06 |
| Waist_Circumference | 8.68095E-06 | 0.000108512 |
| Hip_Circumference | 0.000356035 | 0.002225217 |
| Waist_Hip_ratio | 0.018131208 | 0.056660025 |
| Fat_mass_perc | 0.004064614 | 0.020323071 |
| Lean_mass_perc | 0.044433743 | 0.105674273 |
| Serum_Fe | 0.113568272 | 0.202800485 |
| Total_protein | 0.911352514 | 0.911352514 |
| Total_bilirrubin | 0.316906557 | 0.391171688 |
| ALT_TGP | 0.022160968 | 0.061558245 |
| AST_TGO | 0.06061788 | 0.12628725 |
| GGT | 0.013671991 | 0.048828539 |
| Cholesterol | 0.891486372 | 0.911352514 |
| HDL | 0.000292633 | 0.002225217 |
| Triglycerides | 0.01196343 | 0.048828539 |
| Glucose | 0.04649668 | 0.105674273 |
| Kcal | 0.364377338 | 0.414065157 |
| Carbohydrate_kcal | 0.132582384 | 0.220970639 |
| Proteins_KCAL | 0.38580867 | 0.41935725 |
| Lipids_KCAL | 0.328584218 | 0.391171688 |
| Simple_carbohydrates_kcal | 0.161010196 | 0.25157843 |
| Complex_carbohydrates_kcal | 0.111694347 | 0.202800485 |
| Dairy_products_kcal | 0.231134108 | 0.308743564 |
| Meat_Kcal | 0.234645108 | 0.308743564 |
| Vegetal_Kcal | 0.192820459 | 0.283559498 |
|  |  |  |

Table S2

| **Supplementary Table 2.** Number of reads obtained from sequencing data analysis | | | | |
| --- | --- | --- | --- | --- |
| Gene region | Raw reads | Trimmed reads | Classified reads | Unclassified reads |
|  |  |  | SILVA |  |
| V3-V4 16S rRNA | 4379541 | 4335021 | 3271641 | 1107900 |
|  |  |  | UNITE |  |
| ITS1 | 3775592 | 3728164 | 1240763 | 2534829 |
|  |  |  |  |  |

Table S3

|  |  |  |  |  |  |  |
| --- | --- | --- | --- | --- | --- | --- |
|  | Healthy-weight |  | Overweight |  | Obese |  |
|  | Media | Standard deviation | Media | Standard deviation | Media | Standard deviation |
| **Phylum** |  |  |  |  |  |  |
| Bacteroidota | 45.17 | 7.84 | 38.79 | 10.56 | 29.95 | 10.80 |
| Bacillota | 48.32 | 7.38 | 50.43 | 10.59 | 60.84 | 13.37 |
| Proteobacteria | 3.28 | 1.99 | 4.81 | 5.65 | 4.36 | 4.95 |
| Verrucomicrobiota | 0.07 | 1.37 | 2.37 | 7.64 | 1.54 | 3.65 |
| Desulfobacterota | 0.00 | 0.00 | 1.59 | 0.82 | 1.61 | 0.92 |
| Actinobacteriota | 0.06 | 0.88 | 1.50 | 1.67 | 0.00 | 0.00 |
| Others | 3.23 |  | 0.51 |  | 1.69 |  |
| **Genus** |  |  |  |  |  |  |
| *Prevotella* | 26.05 | 14.41 | 9.87 | 8.29 | 4.51 | 6.42 |
| *Subdoligranulum* | 1.70 | 1.16 | 3.66 | 2.78 | 7.61 | 6.30 |
| *Bacteroides* | 15.52 | 5.96 | 18.88 | 6.79 | 20.51 | 8.79 |
| *Dialister* | 5.68 | 8.00 | 8.45 | 9.78 | 8.16 | 9.36 |
| *Shigella* | 1.42 | 1.34 | 3.15 | 3.85 | 3.39 | 4.56 |
| *Parabacteroides* | 3.19 | 2.44 | 4.41 | 3.10 | 2.87 | 1.77 |
| *Agathobacter* | 2.11 | 1.21 | 3.42 | 3.29 | 3.26 | 2.48 |
| *Phascolarctobacterium* | 6.39 | 4.51 | 6.08 | 5.28 | 7.50 | 7.66 |
| *Ruminococcus* | 2.99 | 1.59 | 3.86 | 2.92 | 4.64 | 4.96 |
| *Alistipes* | 3.94 | 2.47 | 4.73 | 3.65 | 3.65 | 2.41 |
| *Akkermansia* | 0.00 | 0.00 | 3.02 | 7.64 | 2.04 | 3.65 |
| *Paraprevotella* | 2.56 | 2.95 | 2.39 | 2.62 | 3.30 | 3.06 |
| *Roseburia* | 1.65 | 1.01 | 1.38 | 1.70 | 4.60 | 3.24 |
| *Lachnospira* | 3.69 | 2.23 | 0.00 | 3.39 | 3.39 | 2.52 |
| *Faecalibacterium* | 5.21 | 2.45 | 1.77 | 0.88 | 4.20 | 2.29 |
| *Acidaminococcus* | 0.00 | 0.00 | 2.47 | 4.08 | 0.00 | 0.00 |
| *Desulfovibrio* | 0.00 | 0.00 | 1.53 | 1.73 | 1.58 | 1.01 |
| *Megasphaera* | 0.00 | 0.00 | 1.08 | 1.80 | 1.96 | 2.94 |
| *Sutterella* | 1.24 | 0.78 | 0.00 | 0.00 | 0.00 | 0.00 |
| *Alloprevotella* | 2.68 | 2.84 | 3.59 | 4.91 | 0.00 | 0.00 |
| *Coprococcus* | 1.85 | 1.46 | 1.19 | 1.07 | 1.46 | 0.91 |
| *Barnesiella* | 1.29 | 1.30 | 1.77 | 1.97 | 1.25 | 1.55 |
| Others | 10.84 |  | 13.31 |  | 10.12 |  |
|  |  |  |  |  |  |  |

Table S4

|  |  |  |  |  |  |  |
| --- | --- | --- | --- | --- | --- | --- |
|  | Healthy-weight |  | Overweight |  | Obese |  |
|  | Media | Standard deviation | Media | Standard deviation | Media | Standard deviation |
| **Phylum** |  |  |  |  |  |  |
| Ascomycota | 97.19 | 9.11 | 94.72 | 7.61 | 93.26 | 6.16 |
| Basidiomycota | 2.81 | 2.47 | 5.27 | 2.98 | 6.74 | 2.09 |
| Others | 0.00 |  | 0.01 |  | 0.00 |  |
| **Genus** |  |  |  |  |  |  |
| *Nakaseomyces* | 41.39 | 32.01 | 17.48 | 26.53 | 28.33 | 23.59 |
| *Saccharomyces* | 9.15 | 25.25 | 36.00 | 42.28 | 44.52 | 40.46 |
| *Kazachstania* | 26.29 | 27.26 | 1.10 | 2.76 | 0.00 | 0.00 |
| *Candida* | 4.15 | 6.70 | 21.86 | 24.01 | 3.35 | 3.43 |
| *Debaryomyces* | 1.15 | 2.85 | 8.64 | 21.37 | 4.96 | 20.28 |
| *Hanseniaspora* | 7.52 | 20.76 | 0.05 | 1.29 | 0.01 | 1.49 |
| *Malassezia* | 1.95 | 6.52 | 4.76 | 7.07 | 6.31 | 6.33 |
| *Kluyveromyces* | 3.07 | 8.76 | 2.71 | 3.02 | 0.06 | 1.61 |
| *Torulaspora* | 1.02 | 3.23 | 0.00 | 0.00 | 0.00 | 0.00 |
| *Saturnispora* | 0.00 | 0.00 | 1.22 | 1.74 | 0.00 | 0.00 |
| *Pichia* | 0.00 | 0.00 | 0.00 | 0.00 | 1.84 | 3.13 |
| *Kodamaea* | 0.00 | 0.00 | 0.00 | 0.00 | 1.27 | 6.91 |
| *Yarrowia* | 0.00 | 0.00 | 0.00 | 0.00 | 1.46 | 2.00 |
| Others | 4.31 |  | 6.23 |  | 7.95 |  |
